# Competitive Interactions between *C*. *albicans*, *C*. *glabrata* and *C*. *krusei* during Biofilm Formation and Development of Experimental Candidiasis

**DOI:** 10.1371/journal.pone.0131700

**Published:** 2015-07-06

**Authors:** Rodnei Dennis Rossoni, Júnia Oliveira Barbosa, Simone Furgeri Godinho Vilela, Jéssica Diane dos Santos, Patrícia Pimentel de Barros, Márcia Cristina de Azevedo Prata, Ana Lia Anbinder, Beth Burgwyn Fuchs, Antonio Olavo Cardoso Jorge, Eleftherios Mylonakis, Juliana Campos Junqueira

**Affiliations:** 1 Department of Biosciences and Oral Diagnosis, Univ Estadual Paulista/UNESP, São José dos Campos, São Paulo, Brazil; 2 Embrapa Gado de Leite, Juiz de Fora, Minas Gerais, Brazil; 3 Division of Infectious Diseases, Rhode Island Hospital, Alpert Medical School of Brown University, Providence, Rhode Island, United States of America; Louisiana State University, UNITED STATES

## Abstract

In this study, we evaluated the interactions between *Candida albicans*, *Candida krusei* and *Candida glabrata* in mixed infections. Initially, these interactions were studied in biofilms formed *in vitro*. CFU/mL values of *C*. *albicans* were lower in mixed biofilms when compared to the single biofilms, verifying 77% and 89% of *C*. *albicans* reduction when this species was associated with *C*. *glabrata* and *C*. *krusei*, respectively. After that, we expanded this study for *in vivo* host models of experimental candidiasis. *G*. *mellonella* larvae were inoculated with monotypic and heterotypic *Candida* suspensions for analysis of survival rate and quantification of fungal cells in the haemolymph. In the groups with single infections, 100% of the larvae died within 18 h after infection with *C*. *albicans*. However, interaction groups achieved 100% mortality after 72 h of infection by *C*. *albicans*-*C*. *glabrata* and 96 h of infection by *C*. *albicans*-*C*. *krusei*. *C*. *albicans* CFU/mL values from larvae hemolymph were lower in the interacting groups compared with the monoespecies group after 12 h of infection. In addition, immunosuppressed mice were also inoculated with monotypic and heterotypic microbial suspensions to induce oral candidiasis. *C*. *albicans* CFU/mL values recovered from oral cavity of mice were higher in the group with single infection by *C*. *albicans* than the groups with mixed infections by *C*. *albicans*-*C*. *glabrata* and *C*. *albicans*-*C*. *krusei*. Moreover, the group with single infection by *C*. *albicans* had a higher degree of hyphae and epithelial changes in the tongue dorsum than the groups with mixed infections. We concluded that single infections by *C*. *albicans* were more harmful for animal models than mixed infections with non-*albicans* species, suggesting that *C*. *albicans* establish competitive interactions with *C*. *krusei* and *C*. *glabrata* during biofilm formation and development of experimental candidiasis.

## Introduction

*Candida albicans* is the leading cause of fungal infections in the oral cavity, representing 50 to 70% of isolated yeast [1–4]. Although *C*. *albicans* is still the most prevailing species, isolation of non-*albicans* species such as *C*. *tropicalis*, *C*. *parapsilosis*, *C*. *krusei*, *C*. *kefyr*, *C*. *glabrata*, *C*. *guilliermondii*, *C*. *dubliniensis* and *C*. *lusitaniae* is gradually increasing [5–7]. Members of our group verified that 42% of HIV positive patients presented mixed oral infection by various *Candida* species, with *C*. *albicans* and *C*. *glabrata*, *C*. *tropicalis* or *C*. *krusei* being the most recurrent associations [8]. Despite an increase in the number of infections by multiple *Candida* species, information is not widely available on the ecological interactions among these species.

Most of the studies of mixed infections have been focused on the interaction between *C*. *albicans* and bacterial species [9–11] and few studies attempted to investigate interactions of *C*. *albicans* with non-*albicans Candida* species. In one of the earliest studies, Kirkpatrick et al. [12] observed that *C*. *albicans* established a competitive interaction with *C*. *dubliniensis* under broth and biofilm growing conditions. Thein et al. [13] showed that high concentrations of *C*. *krusei* suppressed *C*. *albicans* populations in biofilms formed on acrylic surfaces. On the other hand, Pereira-Cenci et al. [14] did not find competitive interaction between *C*. *albicans* and *C*. *glabrata* in biofilms grown on various surfaces of dental materials. More recently, Alves et al. [15] investigated the *in vitro* co-infection of a reconstituted human vaginal epithelium by *C*. *albicans* and *C*. *glabrata* and observed higher tissue damage in co-infection compared to single *C*. *albicans* infection, suggesting a potential synergism between these species. Since all these studies were performed using *in vitro* model systems, additional *in vivo* studies are required to better understand the interaction of *Candida* species during the colonization and invasion of host tissues in mixed infection.

Animal models are important tools for assessing pathogenicity of *Candida* species [16–18]. An animal host model can mimic the pathogenesis of infection in humans, including colonization, invasion and interaction with the host’s immune system [16,19]. In recent decades, a large number of invertebrate models, including *Galleria mellonella*, have been developed and are being used to study of experimental pathogenicity. These models provide studies on large scale, serving as screening for studies on vertebrate animals, such as mice and rats [17, 20].

Due to the high incidence of candidiasis lesions in immunocompromised patients and the emergence of *non-albicans* species, which are often isolated in association with *C*. *albicans* in mixed infections, the objective of this study was to evaluate the interactions of *C*. *albicans* with *C*. *krusei* and *C*. *glabrata* in biofilms formed *in vitro* and to expand these findings for *in vivo* models of invertebrates and immunosuppressed mice.

## Materials and Methods

### Microbial strains and culture conditions

The *Candida* strains used in this study were *C*. *albicans* (ATCC 18804), *C*. *krusei* (ATCC 6258) and *C*. *glabrata* (ATCC 9030). Strains were stored as frozen stocks with 20% glycerol at -80°C and subcultured on Sabouraud Dextrose Agar (SDA) plates at 37°C. Strains were routinely grown in Yeast Nitrogen Base (YNB, Himedia, Mumbai, India) liquid medium at 37°C in a shaking incubator.

### Fungal inoculum preparation

*C*. *albicans*, *C*. *krusei* and *C*. *glabrata* cells were grown on Sabouraud Dextrose Agar (Himedia, Mumbai, India) for 24h at 37°C. Next, each strain was grown in Yeast Nitrogen Base liquid medium (Himedia, Mumbai, India) with 100 μM of glucose added (Vetec, Rio de Janeiro, Brazil) and then incubated at 37°C for 18 h. Cells were collected by centrifugation and washed three times with PBS. Yeast cells were counted using a hemocytometer. The cell number was confirmed by determining CFU/mL on SDA plates.

### Microbial interactions in biofilms formed at the bottom of 96-well plates: Quantification of CFU/mL

The biofilms were formed following a methodology described by Seneviratne et al. [21] with a few modifications. At first, standardized suspensions of each *Candida* species were adjusted to 10^7^ cells/mL. For mixed biofilms, 100 μL of each species of *Candida* were pipetted onto 96 wells microtiter plates (Costar Corning, New York, USA). To form the single biofilms, 100 μL or 200 μL of each species of *Candida* were added. The plates were incubated at 75 rpm shaking incubator (Quimis, Diadema, São Paulo) at 37°C for 90 min in order to establish the initial stages of cell adhesion. Following this step, the *Candida* suspension was carefully removed by suction, and each well was washed with 200 μL of PBS. This procedure was repeated twice to remove non-adhered cells. Subsequently 200 μL of YNB with 100 μM of glucose (Difco, Detroit, USA) were pipetted, with the plates being incubated at 37°C for 48 h in a shaking incubator (Quimis, Diadema, Sao Paulo). The liquid medium was replaced after 24 h.

After 48 h, the contents of the plate containing the formed biofilms were removed by suction and washed 4 times with PBS. Next, 200 μL of PBS were inserted into each plate well. The biofilm formed at the bottom of the 96 wells microtiter plate (Costar Corning, New York, USA) was carefully scraped with a sterile wooden stick. Subsequently, 100 μL were removed by suction from each well and transferred to a falcon tube containing PBS.

To disaggregate the biofilm, tubes underwent homogenization for 30 s using a 50 W ultrasonic homogenizer (Sonoplus HD 2200—Bandelin Eletronic). From the solution containing PBS and the detached biofilm, a series of dilutions were made up to 10^-4^. Aliquots of 0.1 mL of each dilution were plated in *Petri* plates containing a chromogenic medium HiCrome *Candida* (Himedia, Mumbai, India). The plates were incubated at 37°C for 48 h. After the incubation period, *Candida* species in heterotypic biofilms were differentiated by colony color in HiCrome *Candida* agar and the number of CFU/mL of each species of *Candida* was quantified. The percentage of CFU/mL reduction for *C*. *albicans* cells was calculated considering the heterotypic groups formed by *C*. *albicans*-*C*. *glabrata* or by *C*. *albicans*-*C*. *krusei* in relation to monotypic biofilms of *C*. *albicans*. For statistical analysis and comparison between the groups, the data of CFU/mL were converted to logarithmic form.

### Microbial interactions in biofilms formed on silicone pads: Quantification of biofilm mass

The biofilms were formed according to the methodology described by Tampakakis et al. [10]. In brief, *C*. *albicans*, *C*. *glabrata* and *C*. *krusei* were grown in YPD medium (2% dextrose, 2% Bacto Peptone, 1% yeast extract) overnight at 30°C and diluted to an OD600 of 0.5 in Spider medium. To form the mixed groups, 1 mL of each *Candida* species was added to a well of a sterile 12-well plate containing a silicone pad measuring 1.5 × 1.5 cm that had been pretreated overnight with bovine serum (Sigma-Aldrich). For single biofilms, 1 mL or 2 mL of each species of *Candida* were added.

The inoculated 12-well plate was incubated with gentle agitation (150 rpm) for 90 min at 37°C for adhesion to occur and the standardized samples were washed with 2 mL PBS, and incubation was continued for 60 h at 37°C at 150 rpm in 2 mL of fresh Spider medium. A negative control group of Spider medium without any fungal cells was also included in this study. The silicone pads with biofilm were removed from the wells, dried overnight, and weighed the following day. The total biomass (mg) of each biofilm was calculated by subtracting the weight of the platform material prior to biofilm growth from the weight after the drying period and adjusting for the weight of a control pad unexposed to cells.

### Microbial interactions in the *Galleria mellonella* model

For this study, the methodologies described by Mylonakis et al. [22] and Cowen et al. [23] were used with some modifications. *Galleria mellonella* (Embrapa Gado de Leite, Juiz de Fora, MG 36038–330, Brazil) in their final larval stage were stored in the dark and used within 7 days from shipment. Sixteen randomly chosen *G*. *mellonella* larvae with similar weight and size (250–350 mg) were used per group in all assays. Two control groups were included in the assays that form part of this study: one group was inoculated with PBS to enable us to observe the demise of the larvae due to physical trauma, and the other received no injection as a control for general viability.

Each of the *Candida* strain suspensions was prepared from cultures in 5 mL of YNB liquid medium at 37°C for 18 h. From that, cells were centrifuged at 2000 Xg for 10 min, and the supernatant discarded. The cell pellet was dissolved in PBS and homogenized in tube shaker for 30 seconds. This cell-cleansing procedure was further repeated twice. Cell densities were adjusted using a hemocytometer.

For mixed infections, the larvae were infected with 1x10^6^ CFU/larva of *C*. *albicans* and 1x10^6^ CFU/larva of non-*albicans* species (*C*. *glabrata* or *C*. *krusei)* in different prolegs. For single infections, the larvae also received two injections: one with 1x10^6^ CFU/larva of *Candida (C*. *albicans*, *C*. *glabrata* or *C*. *krusei)* and other with PBS. In addition, another group of single infection by *C*. *albicans* was made, in which the larvae were infected with 2x10^6^ CFU/larva.

The *Candida* suspensions were injected into the haemolymph of each larva via last two prolegs, using a Hamilton 10 μL syringe. Cell densities of *Candida* inoculum were confirmed by CFU/mL measurements in Sabouraud Dextrose Agar.

### Survival curve analysis

Following inoculation, the larvae were stored in plastic containers at 37°C and the number of *G*. *mellonella* killed was recorded daily for a period of 5 days. The larvae were considered dead when they did not react to touch.

### *Candida* CFU/mL quantification in *G*. *mellonella* hemolymph

In order to quantify the number of each species of *Candida* in *G*. *mellonella* infections, fungal cells were extracted from *G*. *mellonella* hemolymph immediately after inoculation (0) and at 4, 8, 12, 18 and 24 h intervals after the larvae were infected with the different species of *Candida*. Five larvae were used in order to collect enough hemolymph. The experiment was performed in triplicate, a total of 15 caterpillars were used for each group and period.

The surviving larvae were cut lengthwise using a scalpel blade, and squeezed in order to retrieve the hemolymph, which were then placed in Eppendorf tubes of approximately 100 μL in volume. Next, the extracted hemolymph was homogenized and serially diluted. Aliquots of 0.1 mL of each dilution were transferred to *Petri* plates containing HiCrome *Candida* chromogenic medium (Himedia, Mumbai, India). The plates were incubated for 48h at 37°C and colonies were subsequently counted to enable the calculation of CFU/mL values.

### Microbial interactions in immunosuppressed mice

The Animal Research Ethics Committee from the Institute of Science and Technology at UNESP, approved this study under protocol number 014/2011 –PA/CEP. Forty adult male mice (*Mus musculus*, Albinus, Swiss), weighing between 30 and 60 g were included in this study. Animals were divided into 5 groups: *C*. *albicans* (n = 8), *C*. *glabrata* (n = 8), *C*. *krusei* (n = 8), *C*. *albicans*-*C*. *glabrata* (n = 8) and *C*. *albicans*-*C*. *krusei* (n = 8). The design of the experiments is shown in Table 1.

**Table 1:** Design of the study of interaction between *Candida* species in the oral candidiasis induced in an immunosuppressed mice model.

Day	Methodology
Day 1	1° injection of prednisolone
Day 2	Inoculation of *Candida* spp. in the oral cavity of mice
Day 3	2° injection of prednisolone
Day 4	Inoculation of *Candida* spp. in the oral cavity of mice
Day 5	3° injection of prednisolone
Day 6	Recovery of *Candida* from the tongue dorsum of mice
	Euthanasia of the mice: macroscopic analysis and optical microscopy of the tongue dorsum of mice

The methodology described by Takakura et al. [24] was used to induce experimental candidiasis with some modifications. In summary, animals were immunosuppressed with 3 subcutaneous injections of prednisolone (Depo-Medrol, Pfizer Laboratories Ltd., Guarulhos, SP, Brazil) at 100 mg/kg of body weight alternated with two inoculations of *Candida*. Tetracycline chloride (Terramicina, Pfizer Laboratories Ltd., Guarulhos, SP, Brazil) was administered in the drinking water of the animals at a concentration of 0.83 mg/mL, beginning 1 day before infection and was maintained throughout the experiment. A 50 μL intramuscular injection of chlorpromazine chloride, equivalent to 10 mg/kg of body weight (Amplictil, Sanofi Aventis, Suzano, SP, Brazil) in each thigh was used to sedate the animals.

Each strain of *Candida* was cultured for 24 h at 37°C on Sabouraud Dextrose Agar (Himedia, Mumbai, Maharashtra, India), and were resuspended in 10 mL of PBS, subsequently being centrifuged at 358 x*g* for 10 minutes. The resulting pellet was resuspended in 10 mL PBS and adjusted to 1x10^8^ cells/mL after counting in a Neubauer chamber (Laboroptik GMBH, Bad Homburg, Germany). A sterile swab (Absorve, Cral, São Paulo, SP, Brazil) soaked in the *Candida* suspension was used to inoculate the sedated mice by rubbing the swab for 3 minutes on the tongue dorsum. For the groups with mixed infections, the same procedure was performed, but the swab was soaked in a standard mixed suspension containing 1x10^8^ cells/mL for each *Candida* species.

In order to confirm the final concentration of each *Candida* species in the swab before the oral inoculation in mice, we performed another experiment for counting CFU/mL of *Candida* cells adhered to the swab. Therefore, after the sterile swab had been soaked in the *Candida* suspension, it was transferred to a falcon tube containing PBS and submitted to ultrasonic homogenizer for 30s. A series of dilutions were made and plated in chromogenic medium HiCrome *Candida* (37°C for 48 h) for quantification of CFU/mL for each *Candida* species.

### Recovery of *C*. *albicans* from the tongue dorsum of mice

Samples from the oral cavity were collected with a swab and placed in a tube containing 0.9 mL of PBS and shaken for 1 minute. Considering that the swab absorbed approximately 0.1 mL of saliva from the oral cavity of mice, this solution was estimated to be a 10^-1^ starting dilution of *Candida* from the soaked swab.

A series of dilutions were subsequently performed and 0.1 mL of each dilution was plated in duplicate onto the surface of plates containing chromogenic HiCrome *Candida* (Himedia, Mumbai, India) in order to differentiate the species recovered. Plates were incubated at 37°C for 48 hours and *Candida* colonies were counted to determine colony-forming units (CFU/mL).

### Euthanasia of the mice

The euthanasia of mice was performed within 2 days after the second inoculation with *Candida*, corresponding to 6 days of experiments. This procedure was performed by administration of an overdose of anesthetic. Tongues were then removed for macroscopic and microscopic analysis.

### Macroscopic analysis of candidiasis on the tongue dorsum of mice

Characteristic lesions of candidiasis on the tongue dorsum were observed using a stereomicroscope (Zeiss, Göttingen, Germany). In order to quantify the number of lesions on each tongue dorsum, scores were assigned from 0 to 4: 0, normal; 1, white patches on less than 20% of the surface; 2, white patches covering between 21% and 90% of the surface; 3, white patches on more than 91% of the surface; and 4, thick white patchy pseudo membranes covering more than 91% of the surface [24].

### Optical microscopy of the tongue dorsum of mice

For the purpose of microscopic analysis of the lesions, the tongues were fixed in 10% formalin for 24 hours. After embedding in paraffin, 5 μm tissue slices were cut and stained with hematoxylin-eosin (HE) and periodic acid-Schiff (PAS). The presence of candidiasis was analyzed using optical microscopy (Olympus, CX41, Tokyo, Japan) at X400 magnification.

Candidiasis lesions were quantified by counting the number of hyphae and epithelial lesions in histological sections stained with PAS and HE, respectively. For each stain, two histological sections were randomly selected for each animal. In each histological section, 23 histologic fields were analyzed in an anteroposterior direction, resulting in a total of 46 histologic fields analysed.

The presence of yeasts and hyphae was quantified according to the methodology of Junqueira et al. [25], attributing the following scores to histologic fields: 1, 1 to 5 yeasts/hyphae; 2, 6 to 15 yeasts/hyphae; 3, 16 to 50 yeasts/hyphae; and 4, more than 50 yeasts/hyphae. For statistical analysis, a median of the scores obtained from the 46 histologic fields was determined per animal. The intensity of the tissue lesions was evaluated by counting the number of histologic fields with the presence of epithelial lesions, such as epithelial hyperplasia, basal cell layer disorganization, exocytosis, spongiosis, loss of filiform papillae, hyperkeratosis and intraepithelial micro abscesses development. The mean of the number of histologic sections with epithelial lesions was determined per animal for statistical analysis.

### Statistical analysis

The CFU/mL data of *in vitro* biofilms, of *Candida* in the hemolymph of *G*. *mellonella* and of experiments in mice were converted to logarithmic values and submitted to ANOVA, Tukey's test or Student *t* test. The results of total biomass of the biofilms were evaluated by ANOVA. The analysis of the survival curve was performed in the Graph Pad Prism software using the Log-rank (Mantel-Cox) test. Kruskal-Wallis and Dunn tests were applied to review scores of macroscopic and histological analysis (*p* ≤ 0.05).

## Results

### Microbial interactions in biofilms formed *in vitro*

**Fig 1:**
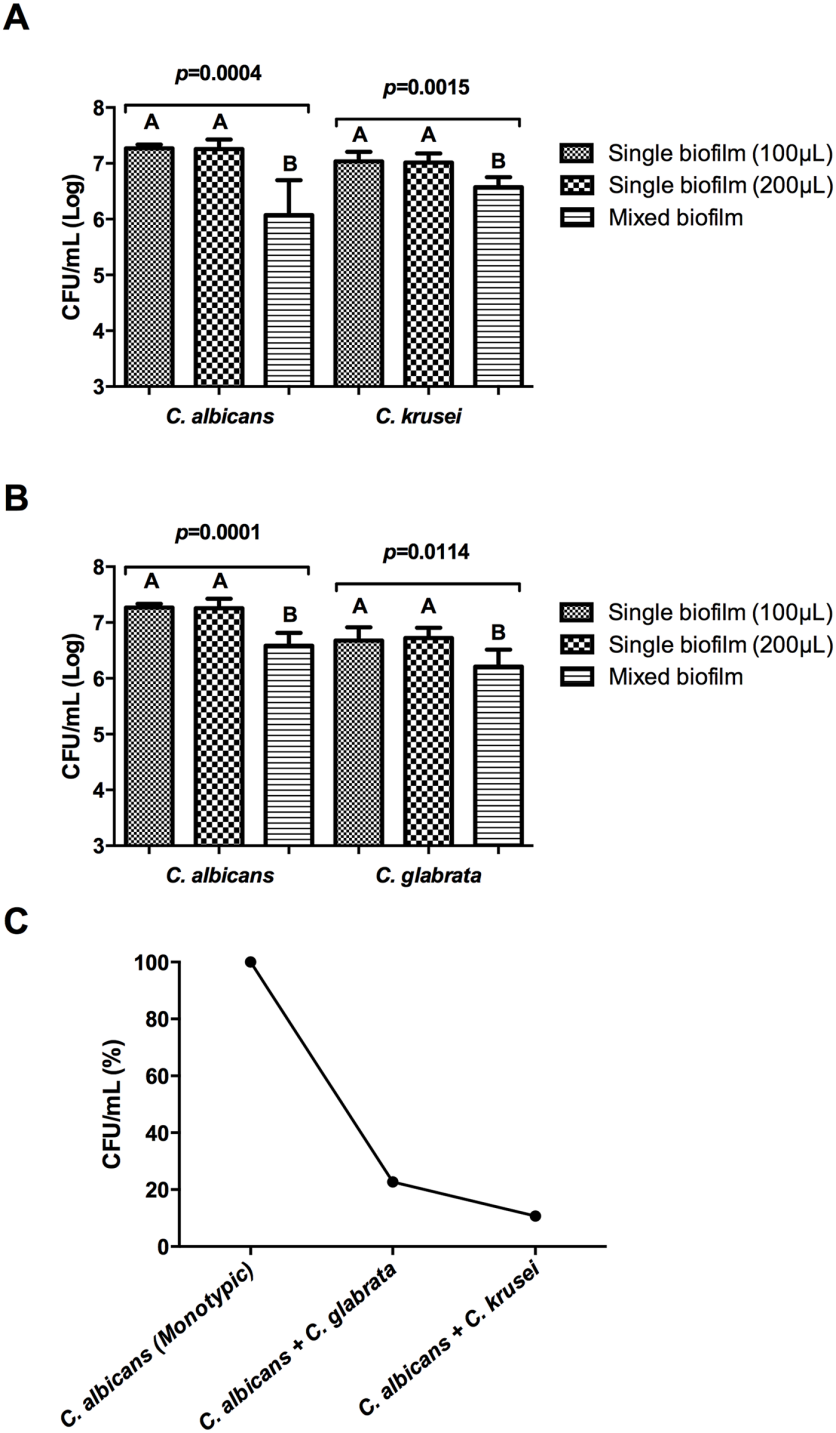
Quantification of cells in biofilms formed at the bottom of 96-well plates (*in vitro* study). (A) Mean and standard deviation of *C*. *albicans* and *C*. *krusei* CFU/mL (Log) values in the following groups: monotypic formed by 200 μL of *C*. *albicans* or *C*. *krusei*; monotypic formed by 100 μL of *C*. *albicans* or *C*. *krusei*; heterotypic formed by 100 μL of *C*. *albicans* and 100 μL of *C*. *krusei*. (B) Mean and standard deviation of *C*. *albicans* and *C*. *glabrata* CFU/mL (Log) values in in the following groups: monotypic formed by 200 μL of *C*. *albicans* or *C*. *glabrata*; monotypic formed by 100 μL of *C*. *albicans* or *C*. *glabrata*; heterotypic formed by 100 μL of *C*. *albicans* and 100 μL of *C*. *glabrata*. Tukey test: different capital letters indicate a significant difference between the three groups analysed. (C) Percentage of reduction, expressed as mean values of CFU/mL, in the viability of *C*. *albicans* when associated with *C*. *glabrata* and *C*. *krusei* relative to monotypic biofilms of *C*. *albicans*.

In biofilms formed at the bottom of 96-well plates, we observed that *C*. *albicans* presented higher CFU/mL in single biofilms compared to mixed biofilms with associations of non-*albicans* species (Fig 1A and 1B), suggesting that *C*. *albicans* establish competitive interactions with *C*. *krusei* and *C*. *glabrata* during biofilm formation. More specifically, when we compared the results obtained from mixed and single biofilms, we found a 77% reduction in *C*. *albicans* CFU/mL values when associated with *C*. *glabrata* opposed to 89% with *C*. *krusei*, demonstrating that the inhibition of *C*. *albicans* was greater in the presence of *C*. *krusei* than in the presence of *C*. *glabrata* (Fig 1C).

**Fig 2:**
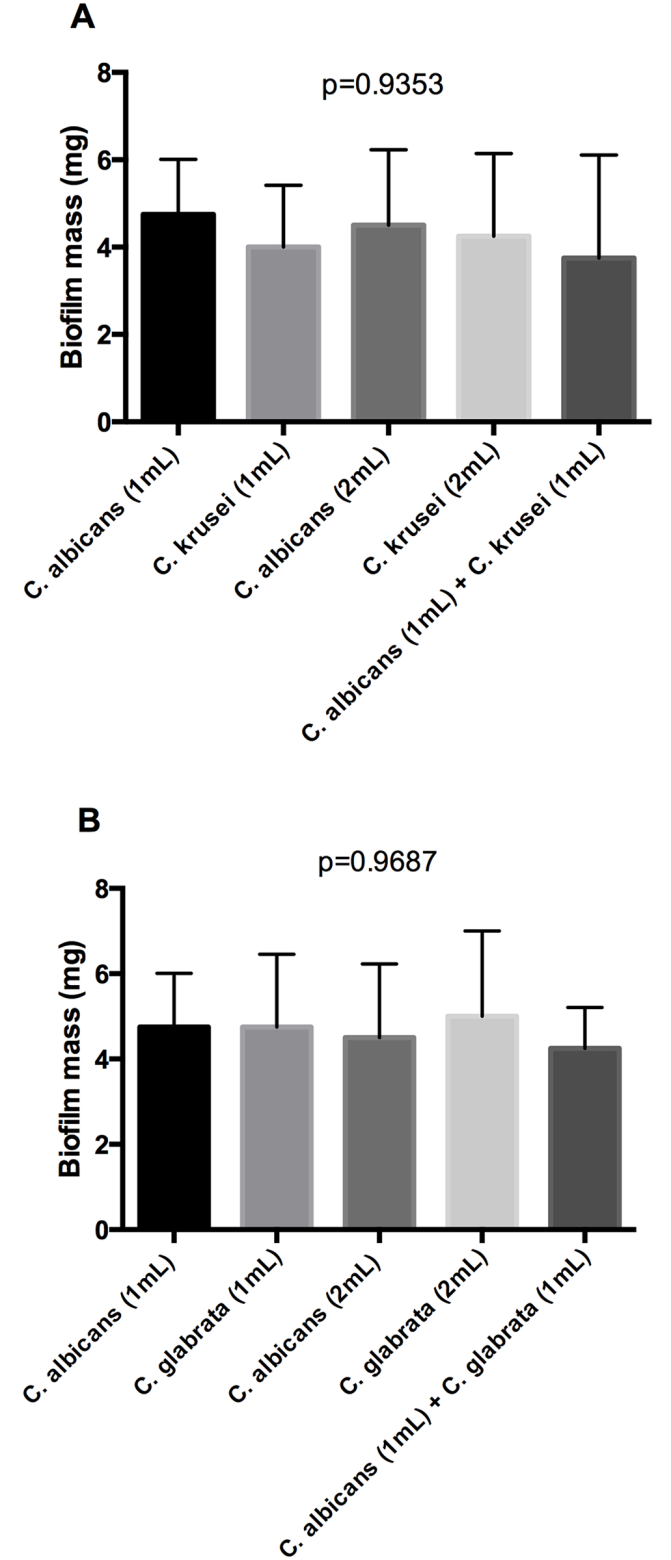
Quantification of the total mass of the biofilm formed on silicone pads (*in vitro* study). (A) Analyse of *C*. *albicans*-*C*. *krusei* interaction. Mean and standard deviation of *Candida* CFU/mL (Log) in the following groups: monotypic formed by 1 mL of *C*. *albicans*; monotypic formed by 1 mL of *C*. *krusei;* monotypic formed by 2 mL of *C*. *albicans*; monotypic formed by 2 mL of *C*. *krusei;* heterotypic formed by 1 mL of *C*. *albicans* and 1 mL of *C*. *krusei*. (B) Analyse of *C*. *albicans*-*C*. *glabrata* interaction. Mean and standard deviation of *Candida* CFU/mL (Log) in the following groups: monotypic formed by 1 mL of *C*. *albicans*; monotypic formed by 1 mL of *C*. *glabrata;* monotypic formed by 2 mL of *C*. *albicans*; monotypic formed by 2 mL of *C*. *glabrata;* heterotypic formed by 1 mL of *C*. *albicans* and 1 mL of *C*. *glabrata*. ANOVA test: there are no statistically significant differences between the groups.

The interaction between different *Candida* species was also evaluated through biofilm growth on silicone pads in order to quantify the total biomass of mixed and single biofilms. The quantity of biofilm mass was similar among all the groups studied (Fig 2), suggesting that the reduction of *C*. *albicans* CFU/mL in mixed biofilms can be attributed to competitive interactions between the *Candida* species for adhesion sites during the initial phase of biofilm formation.

### Microbial interactions in the *G*. *mellonella* model

**Fig 3:**
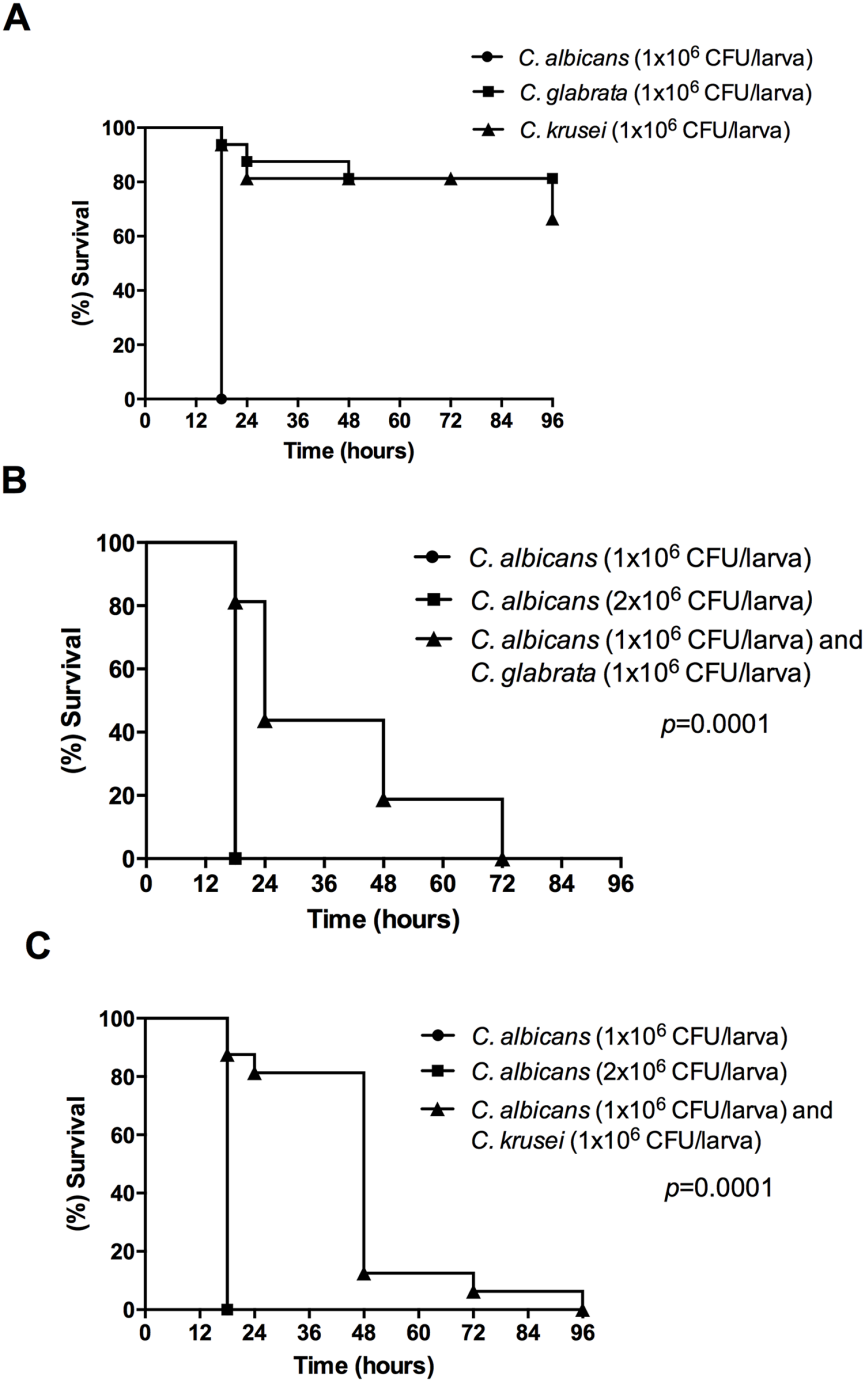
Survival curve of *G*. *mellonella* larvae infected with *Candida* strains. (A) The larvae were infected with *C*. *albicans*, *C*. *krusei* or *C*. *glabrata* (single infections); (B) The larvae were infected with *C*. *albicans* (single infection) and compared with larvae infected by *C*. *albicans* and *C*. *glabrata* (mixed infection); (C) The larvae were infected with *C*. *albicans* (single infection) and compared with larvae infected by *C*. *albicans* and *C*. *krusei* (mixed infection). Comparison of survival curves was made by Log rank test. These experiments were repeated at least twice and representative experiments are presented.

Initially, we tested the pathogenicity of each *Candida* species in the *G*. *mellonella* model. Suspensions of *C*. *albicans*, *C*. *krusei* or *C*. *glabrata* were inoculated into *G*. *mellonella* larvae at a concentration of 1x10^6^ CFU/larva (Single infections). Larvae infected with *C*. *albicans* presented 100% mortality following a period of 18 h post infection, whilst *C*. *krusei* and *C*. *glabrata* species were less pathogenic to the *G*. *mellonella* caterpillars, with a mortality rate of 34% and 19%, respectively, at the end of the experiment (Fig 3A).

In order to study the interaction of *Candida* species in the *G*. *mellonella* model, we infected *G*. *mellonella* larvae with 1x10^6^ CFU/larva of *C*. *albicans* and 1x10^6^ CFU/larva of *C*. *krusei* or *C*. *glabrata* (Mixed infections). A significant increase in the *G*. *mellonella* survival rate was observed among those larvae infected by heterotypic suspensions, when compared with the groups infected with *C*. *albicans* monotypic suspensions. The single infection by *C*. *albicans* achieved 100% of mortality when the larvae were inoculated with 2x10^6^ CFU/larva or 1x10^6^ CFU/larva of *C*. *albicans* (Fig 3B and 3C).

**Fig 4:**
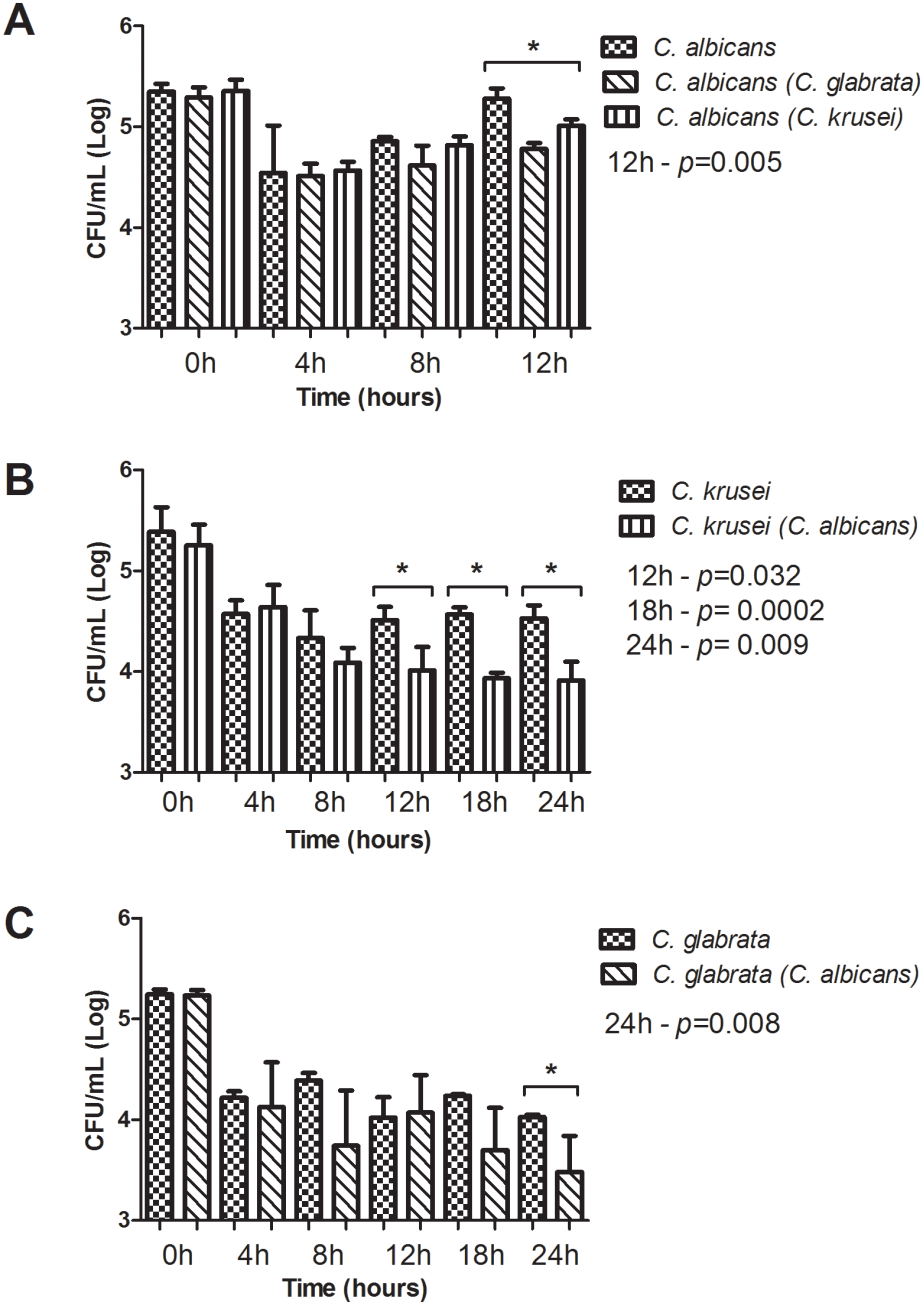
Quantification of fungal cells in *G*. *mellonella* hemolymph. Mean and standard deviation of CFU/mL (Log) values in *G*. *mellonella* hemolymph at various time periods of experimental infection. (A) *C*. *albicans* CFU/mL measurements: significant statistical differences were observed between monotypic and heterotypic groups in the 12 h period (ANOVA and Tukey’s test). *C*. *albicans* was not quantified in *G*. *mellonella* hemolymph at 18 and 24 h because all larvae had perished by those times. (B) *C*. *krusei* CFU/mL measurements: significant statistical differences were observed between groups in the 12, 18 and 24 h periods (*Student t* test). (C) *C*. *glabrata* CFU/mL measurements: significant statistical differences were observed between monotypic and heterotypic groups in the 24 h period (*Student t* test).

*Candida* CFU/mL measurements of *G*. *mellonella* hemolymph displayed similar growth patterns in the infection by *C*. *albicans* in both single and mixed infections at 0, 4 and 8 hours after fungal inoculation. However, a significant statistical difference between the groups was found at 12h post-infection (Fig 4A), where *C*. *albicans* presented noticeably poorer growth in the interaction group when compared to the monotypic group.

The CFU/mL number of *C*. *krusei* and *C*. *glabrata* in the mixed infections were also counted, verifying that *C*. *krusei* presented considerably lesser growth in the interaction group when compared to the monotypic group at 12, 18 and 24h (Fig 4B). *C*. *glabrata* showed similar growth patterns in both single and mixed infections at 0, 4, 8, 12, and 18h, although at 24 h after infection, a significant statistical difference was observed between the groups (Fig 4C).

According to these results, the survival curves analysed and the CFU/mL values obtained, it is posible to confirm that mixed infections groups presented a lower infection rate in *G*. *mellonella* when compared to the groups with single infections by *C*. *albicans*, indicating again a competitive interaction between species in *C*. *albicans—C*. *krusei* and *C*. *albicans—C*. *glabrata* interactions.

### Microbial interactions in immunosuppressed mice

**Fig 5:**
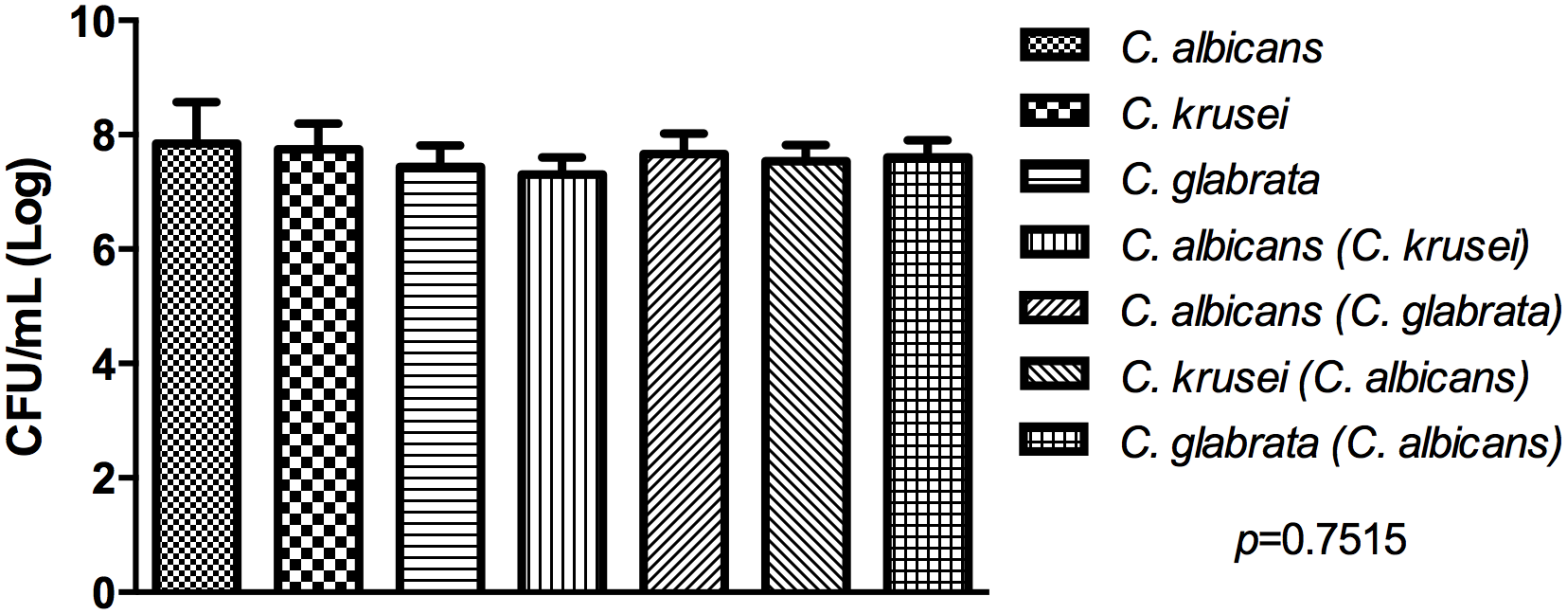
Quantification of *Candida* cells adhered to the swab. Mean and standard deviation of the CFU/mL (Log) of *C*. *albicans* in monotypic suspension, *C*. *krusei* in monotypic suspension, *C*. *glabrata* in monotypic suspension, *C*. *albicans* in heteroptypic suspension with *C*. *krusei*, *C*. *albicans* in heteroptypic suspension with *C*. *glabrata*, *C*. *krusei* in heteroptypic suspension with *C*. *albicans*, and *C*. *glabrata* in heteroptypic suspension with *C*. *albicans*. ANOVA test: there are no statistically significant differences between the groups.

The interaction *Candida* study established in *G*. *mellonella* larvae was moved to a model of experimental oral candidiasis in immunosuppressed mice. Monotypic and heterotypic suspensions of *Candida* species were inoculated in the oral cavity of mice using a swab previously soaked in the *Candida* suspensions. Initialy, we did an experiment to confirm the concentration of *Candida* cells adhered to the swab. *C*. *albicans*, *C*. *krusei* and *C*. *glabrata* showed the same capacity to adhere to the swab when they were soaked in both monotypic and heterotypic suspensions (Fig 5).

After *Candida* inoculations, the colonization of each *Candida* species in the mice oral cavity was estimated. Samples from the oral cavity were collected and plated to counting CFU/mL number. In the groups with single infections, we found values ranging from 5.75 ± 0.31 CFU/mL (Log) for *C*. *albicans*, 6.18 ± 0.06 CFU/mL (Log) for *C*. *krusei* and 4.33 ± 0.18 CFU/mL (Log) for *C*. *glabrata* inoculations. The data indicates that all the species studied were capable to grow and colonize the oral cavity of immunosuppressed mice.

**Fig 6:**
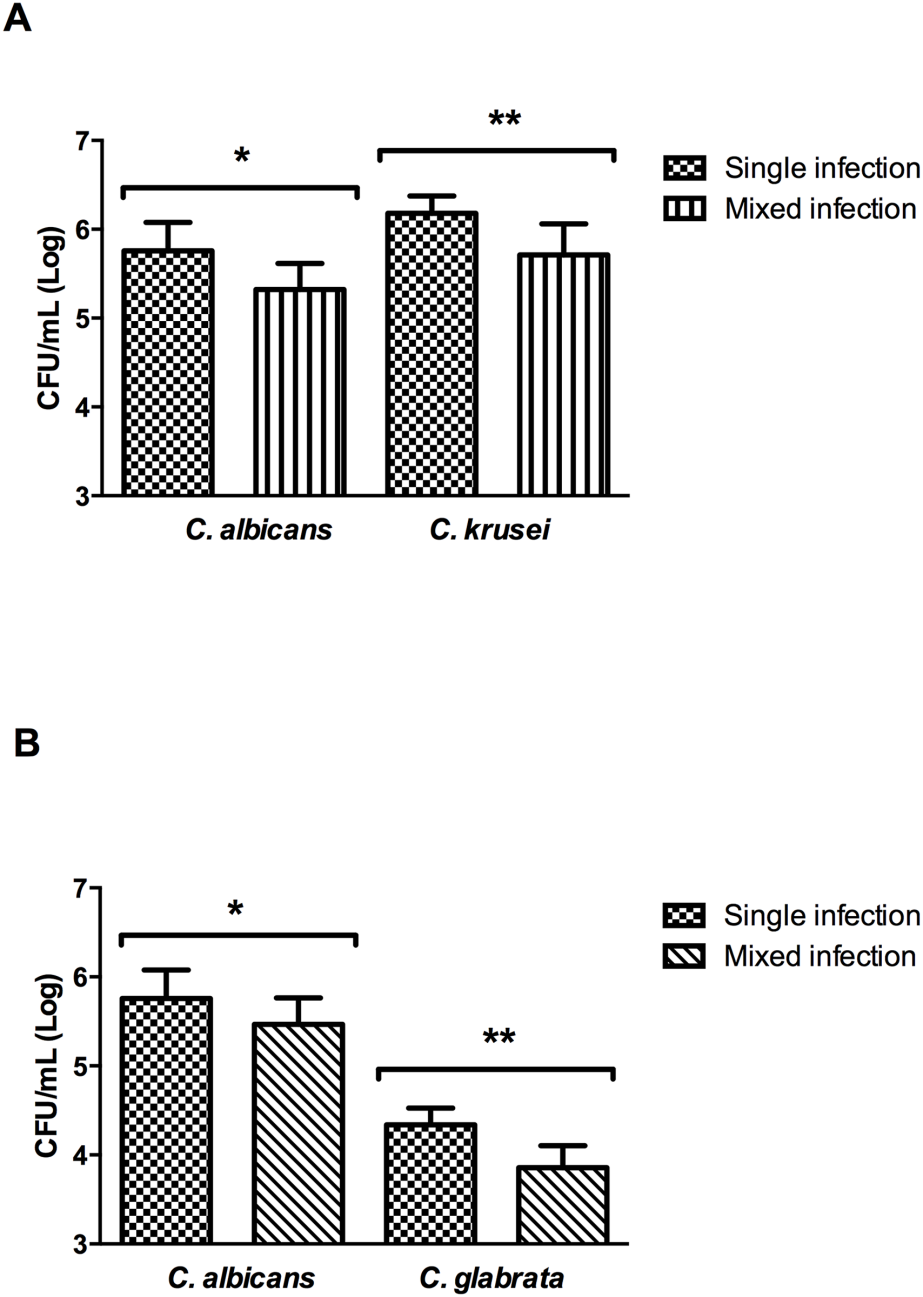
Quantification of fungal cells recovered from the buccal cavity of mice. (A) Mean and standard deviation of the CFU/mL (Log) of *C*. *albicans* and *C*. *krusei* recovered from the buccal cavity of immunosuppressed mice with single and mixed infections. Student *t* test. *CFU/mL of *C*. *albicans*: comparison between single infection by *C*. *albicans* and mixed infection by *C*. *albicans*-*C*. *krusei* (*p* = 0.012); **CFU/mL of *C*. *krusei*: comparison between single infection by *C*. *krusei* and mixed infection by *C*. *albicans*-*C*. *krusei* (*p* = 0.004). (B) Mean and standard deviation of the CFU/mL (Log) of *C*. *albicans* and *C*. *glabrata* recovered from the buccal cavity of immunosuppressed mice with monotypic and heterotypic infections. Student *t* test. *CFU/mL of *C*. *albicans*: comparison between single infection by *C*. *albicans* and mixed infection by *C*. *albicans*-*C*. *glabrata* (*p* = 0.079); **CFU/mL of *C*. *glabrata*: comparison between single infection by *C*. *glabrata* and mixed infection by *C*. *albicans*-*C*. *glabrata* (*p* = 0.001).

Analyzing the *C*. *albicans* colonization profile in single and mixed infections, we observed that CFU/mL values were lower in both mixed infections, with 5.32 log_10_ for association with *C*. *krusei* (*p* = 0.012), and 5.46 log_10_ for association with *C*. *glabrata* (*p* = 0.079), when compared to single infection (5.75 ± 0.31). However, there was only a significant statistical in the association of *C*. *albicans* with *C*. *krusei* (Fig 6). Non-*albicans* species also presented reduction of CFU/mL number in mixed infections compared to single infections. *C*. *krusei* growth was observed as 6.18 log_10_ in single infection and 5.71 log_10_ in mixed infection (*p* = 0.004) (Fig 6A). *C*. *glabrata* growth was observed as 4.33 log_10_ in single infection and 3.85 log_10_ in mixed infection (*p* = 0.001) (Fig 6B).

**Fig 7:**
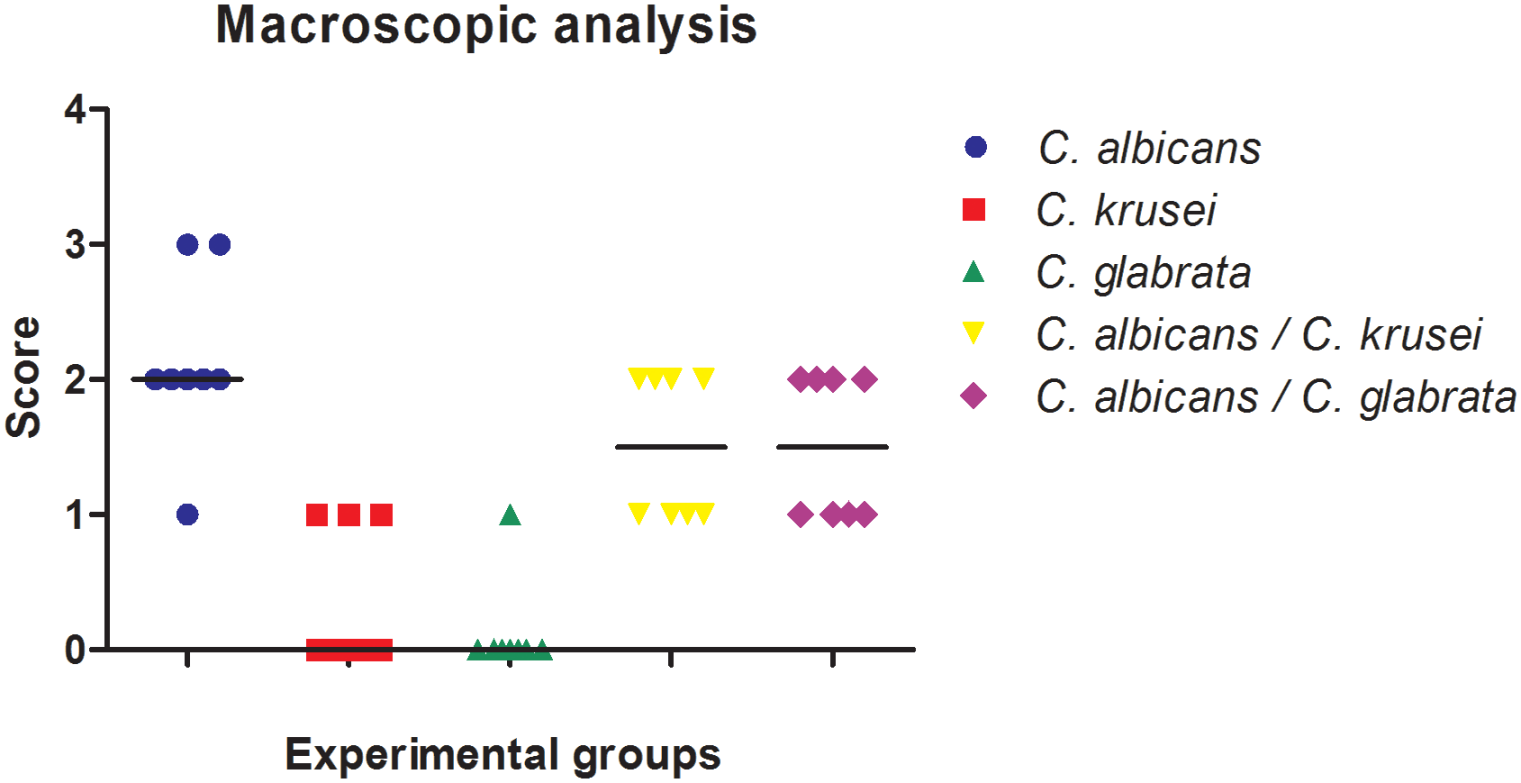
Macroscopic analysis of candidiasis lesions. Scores and medians obtained from the macroscopic examination of the tongue dorsum of groups infected with *C*. *albicans* monospecies, *C*. *krusei* monospecies, *C*. *glabrata* monospecies, *C*. *albicans*-*C*. *krusei* multi-species and *C*. *albicans*-*C*. *glabrata* multi-species. Kruskal-Wallis and Dunn test: significant statistical differences were confirmed between the groups (*p* = 0.0001), with similarities between *C*. *albicans* monospecies and multi-species groups, and variations when compared with *C*. *krusei* and *C*. *glabrata* monospecies groups.

In order to evaluate the development of oral candidiasis in mice, the clinical aspect of tongue dorsum was analysed during the experiments. In the macroscopic analysis of mice with single infection, the animals infected with *C*. *albicans* presented lesions typical of candidosis on their tongue dorsum. These lesions were characterized by white patches with areas of papillary atrophy and presence of pseudomembrane. On the other hand, monospecies groups formed by *C*. *krusei* or *C*. *glabrata* did not present macroscopic candidosis lesions on the tongue dorsum, suggesting that non-*albicans* species were not able to instigate the development such lesions, despite being capable of colonizing the oral cavities of mice. These macroscopic lesions were quantified and we observed that the group with single infection by *C*. *albicans* had a higher median than the groups infected with mixed species, although no significant statistical difference was found between groups (Fig 7).

**Fig 8:**
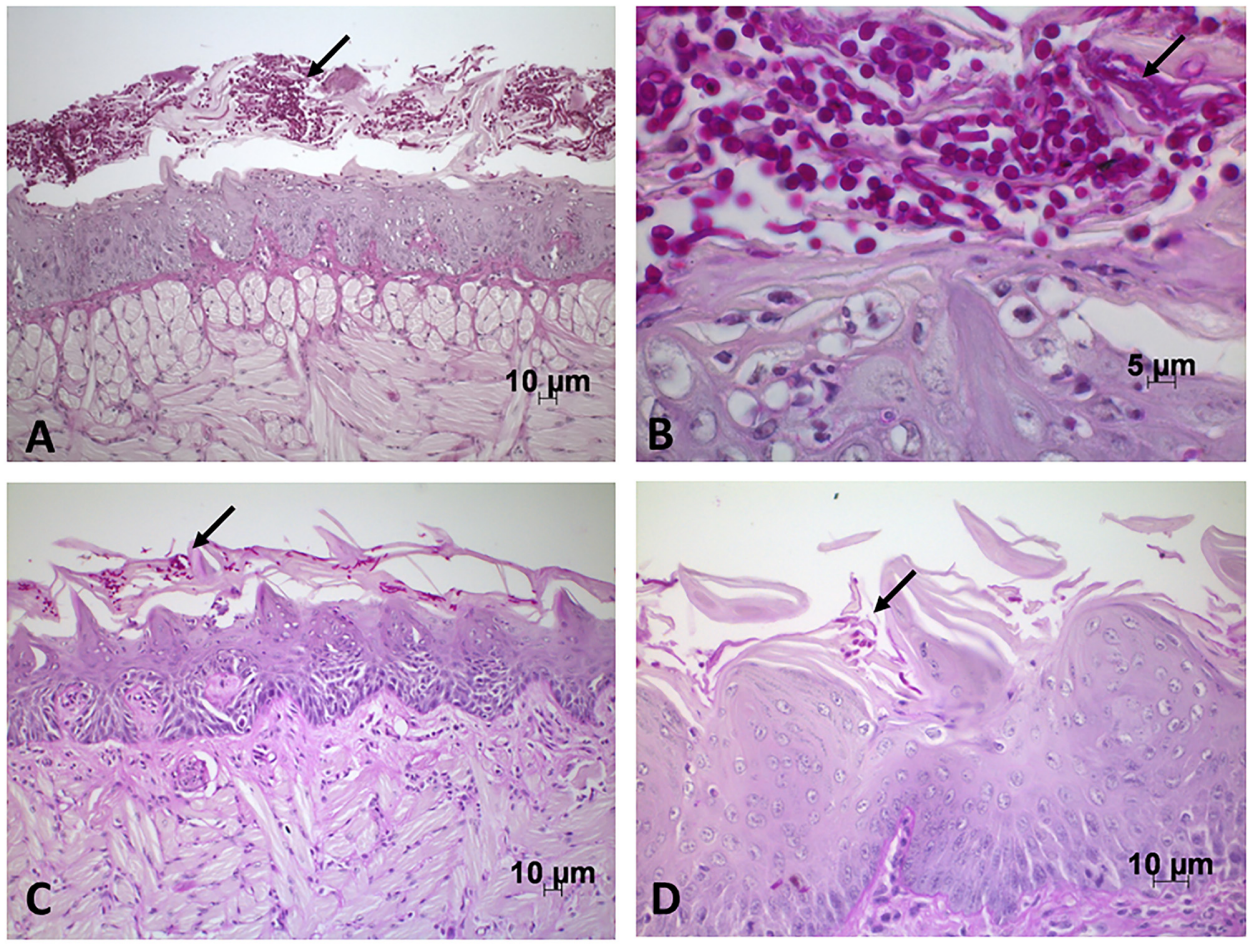
*Candida* hyphae and yeast on the tongue dorsum of mice. (A) Sagittal incision of the tongue dorsum of mice infected with *C*. *albicans* monospecies inoculum, displaying the presence of yeast and hyphae (↓) in keratin. PAS; original magnification: 200X. (B) Sagittal incision of the tongue dorsum of mice infected with *C*. *albicans* monospecies inoculum, displaying the presence of yeast and hyphae (↓) in keratin, and polymorphonuclear leukocytes forming intraepithelial microabscesses. PAS; magnification: 630X. C) Sagittal incision of the tongue dorsum of mice infected with *C*. *albicans* and *C*. *krusei* multi-species inoculum, displaying the presence of yeast and hyphae (↓) in keratin, and spongiosis in the epithelium. PAS; magnification: 200X. D) Sagittal incision of the tongue dorsum of mice infected with *C*. *albicans* and *C*. *glabrata* multi-species inoculum, displaying the presence of yeast and hyphae (↓) in keratin and in the epithelium. PAS; magnification: 400X.

**Fig 9:**
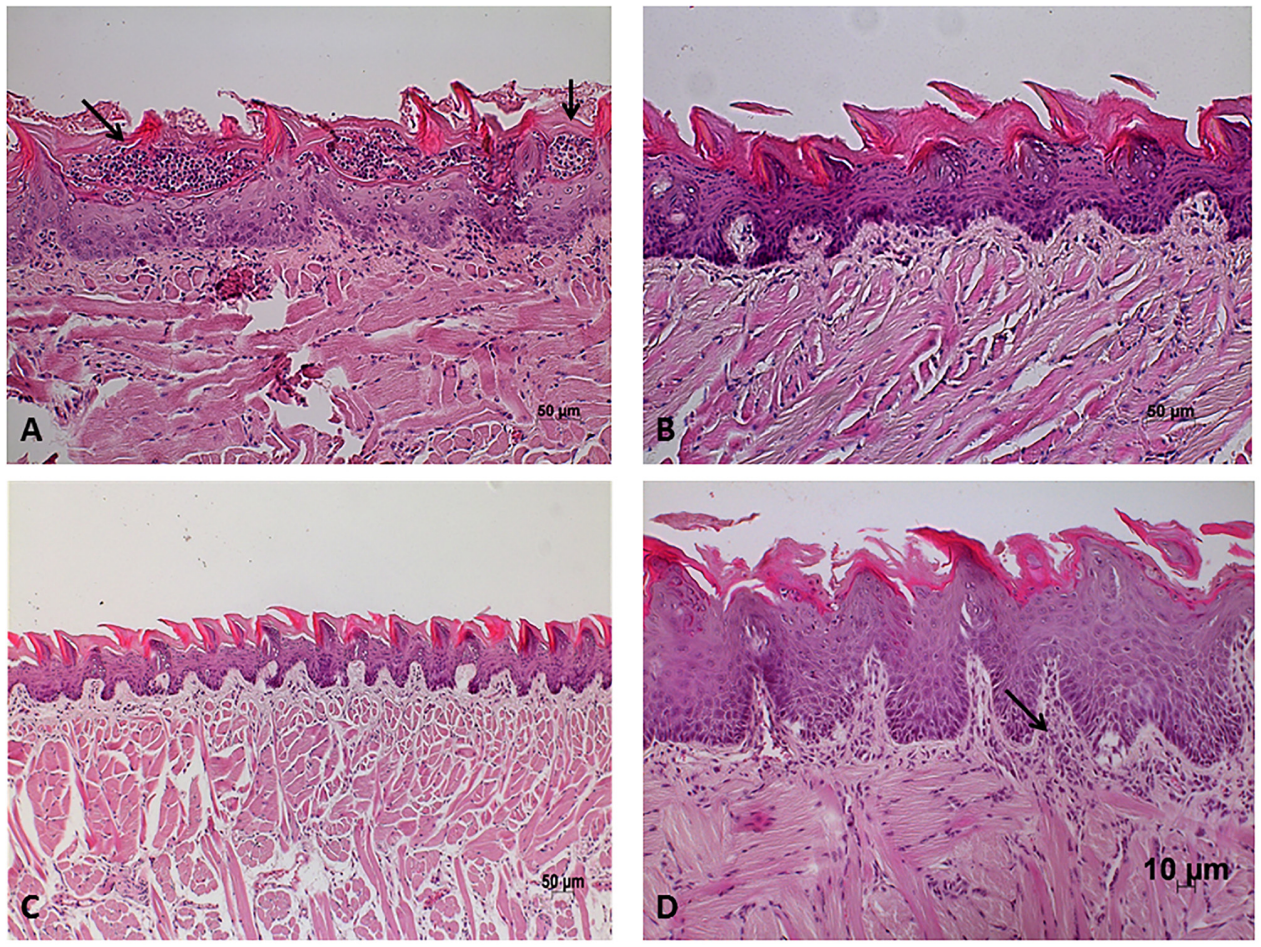
Epithelial changes caused by candidiasis lesions. (A) Sagittal incision of the tongue dorsum of mice infected with *C*. *albicans* monospecies inoculum, displaying the presence of intraepithelial microabscesses (↓), exocytosis and spongiosis. Moderate inflammatory infiltrate is seen in lamina propria. HE; magnification: 200X. (B) Sagittal incision of the tongue dorsum of mice infected with *C*. *krusei* monospecies inoculum, displaying normal tissue appearance. HE; magnification: 200X. (C) Sagittal incision of the tongue dorsum of mice in the *C*. *glabrata* control group, displaying normal tissue appearance. HE; magnification: 100X. (D) Sagittal incision of the tongue dorsum of mice in the *C*. *albicans* and *C*. *krusei* interaction group, displaying the presence of spongiosis, basal layer duplication, hyperplasia and epithelial desquamation. Moderate inflammatory infiltrate is seen in lamina propria (↓). HE; magnification: 200X.

After that, the tongues were submitted to microscopic analysis and we verified the presence of yeast and hyphae in the keratinized layer of the tongue dorsum, specifically concentrated on conical papillae. In the areas with yeast and hyphae, we also found polymorphonuclear leukocytes forming intraepithelial microabscesses and cells of inflammatory infiltrate in the connective tissue (Fig 8). Several lesions were also observed in the epithelium such as desquamation, loss of filiform papillae, loss of stratification, epithelial hyperplasia, exocytosis, spongiosis, acantholysis, hyperkeratosis and basal layer (Fig 9).

**Fig 10:**
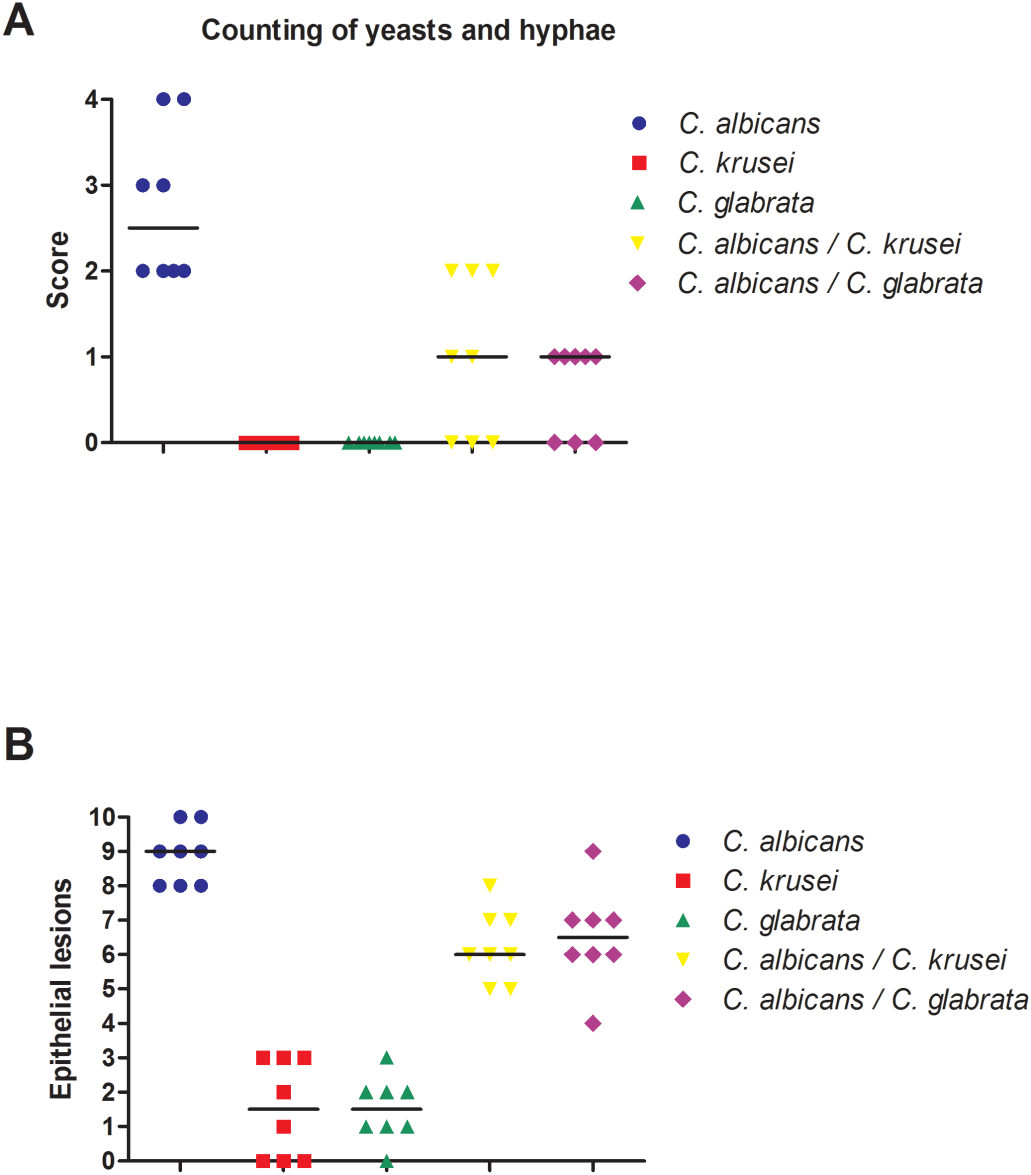
Quantification of hyphae, yeast and epithelial changes. (A) Scores and medians of the number of hyphae and yeast derived from single and mixed infection. Kruskal-Wallis and Dunn test: significant statistical differences were confirmed between the groups (*p* = 0.0009), with variations between *C*. *albicans* monospecies and multi-species groups, and similarities amid the latter. (B) Number of epithelial changes and medians observed in candidosis microscopic lesions on the tongue dorsum of mice inoculated with *Candida* monospecies and multi-species suspensions. Kruskal-Wallis and Dunn test: significant statistical differences were confirmed between the groups (*p* = 0.0013), with variations between *C*. *albicans* monospecies and multi-species groups, and similarities amid the latter.

In the analysis for quantification of yeasts/hyphae and epithelium alterations, the group with single infection by *C*. *albicans* obtained higher medians than the groups with mixed infection formed by *C*. *albicans*-*C*. *krusei* or by *C*. *albicans*-*C*. *glabrata*. In contrast, yeasts and hyphae were not found in the groups inoculated with monotypic suspensions of *C*. *krusei* or *C*. *glabrata*. Moreover, few epithelium alterations were found in these groups. These findings are consistent with the macroscopic results obtained in this study, which demonstrated that these non-*albicans* species are not capable of causing infection in the proposed model, being only able to colonize the oral cavity of mice (Fig 10).

Considering the results observed in immunosuppressed mice, we confirm that *C*. *albicans* compete with non-*albicans* species during the development of experimental oral candidiasis in mixed infections. Single infections by *C*. *albicans* were more harmful for oral cavity of mice than mixed infections with non-*albicans* species.

## Discussion

In this report, we evaluated the interactions between *C*. *albicans*, *C*. *krusei* and *C*. *glabrata* in mixed infections using *in vitro* and *in vivo* study models. In the *in vitro* study, *C*. *albicans* showed lower number of CFU/mL in mixed biofilms with *C*. *glabrata* or *C*. *krusei* when compared to single biofilm formed only by *C*. *albicans*. After that, we expanded this study for *in vivo* models of experimental candidiasis using *G*. *mellonella* and immunosuppressed mice as a host models. In both models, we found that single infections by *C*. *albicans* were more harmful for animals than mixed infections with *C*. *albicans*-*C*. *glabrata* or *C*. *albicans*-*C*. *krusei*.

The reduction of CFU/mL of *C*. *albicans* observed in mixed biofilms in relation to single biofilm suggests that *C*. *albicans* establish competitive interactions with *non- albicans Candida* species during biofilm formation. Kirkpatrick et al. [12] also found competitive interaction between *C*. *albicans* and *C*. *dubliniensis* under broth and biofilm growing conditions. However, Silva et al. [26] found a neutral interaction between *C*. *albicans* and *C*. *glabrata* in biofilms formed on acrylic resine, where the number of each species’ cultivable cells was not affected by each species’ presence.

The *C*. *albicans* reductions observed in mixed biofilms were approximately of 77% for association with *C*. *glabrata* and 89% for interaction with *C*. *krusei*, demonstrating that the inhibition of *C*. *albicans* was greater in the presence of *C*. *krusei* than in the presence of *C*. *glabrata*. Pathak et al. [27] also confirmed slower methabolic activity in multi-species biofilms formed by the association of *C*. *albicans* and *C*. *krusei* compared with biofilms formed by the association of *C*. *albicans* and C. *glabrata*. Our results obtained from mixed biofilms with *C*. *albicans* and *C*. *krusei* are consistent with the study of Thein et al. [13], where a 85% growth reduction in *C*. *albicans* was determined during the interactions between *C*. *albicans* and *C*. *krusei*, in biofilms formed *in vitro*. For these authors, the suppression of *C*. *albicans* in co-inhabition with *C*. *krusei* could be due to the competitive adhesion inhibition of *C*. *albicans* by *C*. *krusei* in the initial phase of biofilm formation or due to chemical or molecular mediators diffusing into the growth medium.

To explore whether competitive interactions between *Candida* species observed in this study can affect the total biomass of mixed biofilms, we utilized the dry weight biofilm assay on silicone pads. This analysis allows us to estimate the quantity of total *Candida* cells and extracellular matrix compared to the viable cells counting method (CFU/mL). In the total biomass quantification, we found no statistically significant difference between single and mixed biofilms, suggesting that the reduction of *C*. *albicans* CFU/mL in mixed biofilms occurred by competition for colonization sites and by nutritional deprivation.

Thus, it is possible to note that the most studies performed *in vitro* found competitive interactions between *C*. *albicans* and non-*albicans Candida* species during biofilm formation. However, there are not sufficient studies done to investigate these interactions during the infection process and the onset of disease. The present study is the first *in vivo* study that attempts to investigate the interactions between the different *Candida* species in the development of experimental candidosis.

In recent decades, a large number of invertebrate models, including *Galleria mellonella*, *Caenorhabditis elegans*, *Drosophila melanogaster and Dictyostelium discoideum*, have been developed and are being used to study of experimental pathogenicity [17, 20]. These models have provided considerable knowledge in different aspects of microbial infection and present numerous advantages compared to mammal models, such as low breeding costs, ease of handling, speed in obtaining results, as well as enabling studies on a large scale, serving as screening for further studies on vertebrates, thereby fulfilling the ethical and legal demands of the 3Rs principle (Reduction, replacement and refinement) [28–30].

Conversely, vertebrate models are extremely important for the study of oral candidosis [31], as these animals simulate similar conditions of those found in the human mouth, such as salivary flow, pH variations, the presence of teeth, characteristics of the mucous membrane and immune system responses [32]. Mice have been a useful experiment model in the development of oral candidosis, since they do not present *Candida spp*. as a microbiota component, are easily obtained in large numbers and low cost maintenance compared with other vertebrates [31, 24, 33–34].

Thereby, we used the invertebrate model of *G*. *mellonella* and the vertebrate model of immunosuppressed mice for the *in vivo* study of experimental candidiasis. For the study in *G*. *mellonella* model, initially we tested the pathogenicity of each *Candida* species in the *G*. *mellonella* larvae. We observed that 100% of larvae infected with *C*. *albicans* monotypic suspensions died after a period of 18h post-infection. *C*. *glabrata* and *C*. *krusei* species were less pathogenic, with mortality rates ranging from 19% to 34%, respectively, 96h after inoculation. These findings are consistent with Cotter et al. [35] and Junqueira et al. [36]. Cotter et al. [35] reported that *C*. *albicans* was the most pathogenic species, causing the death of 90% of larvae, whilst the mortality rate for other species were: 70% for *C*. *tropicalis*, 45% for *C*. *parapsilosis*, 20% for *C*. *krusei* and 0% for *C*. *glabrata*. Junqueira et al. [36] found mortality rates in *G*. *mellonella* equivalent to 100% for *C*. *albicans*, 25% for *C*. *krusei* and 20% for *C*. *glabrata*. Scorzoni et al. [37] reported that in order to obtain a lethal infection with *C*. *krusei* in *G*. *mellonella* it was necessary to inject a yeast concentration 10 times higher than that of *C*. *albicans*. According to these data, we can confirm that non-*albicans* species present lower pathogenicity in the *G*. *mellonella* model compared to *C*. *albicans* species.

An increase in the survival rate of *G*. *mellonella* was observed in the interaction groups, in which *Candida* mixed infections were performed, in contrast with the groups infected with *C*. *albicans* monospecies. *C*. *albicans* single infection achieved 100% mortality at 18h post-infection. On the other hand, mortality rates of 100% were reached 96h after mixed infections with *C*. *albicans* and *C*. *krusei*, and 72h after mixed infections with *C*. *albicans* and *C*. *glabrata*. These findings suggest that during infections in *G*. *mellonella* there is a competitive interaction between *C*. *albicans* and the non-*albicans* species studied, which is consistent with the results obtained in biofilms formed *in vitro* here presented.

Regarding the quantification of *C*. *albicans* CFU/mL in *G*. *mellonella* hemolymph in single infections, we verified that the amount of yeast recovered immediately after inoculation (time 0) was no different from the amount used to initiate the infection. However, a reduction on the number of recovered cells, probably due to the host immune response, was found 4h following inoculation. After this period, it is believed that the caterpillar immune system was not able to fight the infection, thus CFU values gradually increased, reaching a peak at 12h post-infection, which could justify the 100% mortality rate found in the 18h period. While non-*albicans* species CFU was measured in the caterpillar’s hemolymph, it was possible to observe that this host immune system managed to reduce and maintain the fungal load after 4h and during all other periods assessed. This fact may explain the lower mortality rate experienced by these species compared to *C*. *albicans* seen in the survival assays. Besides the increase of *C*. *albicans* cells in the hemolymph, possibly the 100% mortality rate was caused by morphological transition of *C*. *albicans*. Vilela et al. [38] verified several clusters of hyphae in the fat body of *G*. *mellonella* larvae after 18h of infection by *C*. *albicans*.

With regards to CFU values in hemolymph of mixed infections, we verified that *C*. *albicans* yeast levels were significantly higher in single infections than those found in mixed infections with *C*. *krusei* or *C*. *glabrata* after a period of 12h post-infection, suggesting that the presence of these non-albicans species in *G*. *mellonella* results in less lethal infections. This would appear to also justify the greater survival rate larvae displayed in the interaction groups compared to the monotypic groups studied.

Considering that single infections by *C*. *albicans* were considerably more pathogenic to *G*. *mellonella* than mixed infections induced by the association of *C*. *albicans* with non-*albicans* species, the present study was expanded to encompass oral candidosis in immunosuppressed mice models. Based on the CFU/mL data analysis of yeast recovered from the buccal cavity of immunosuppressed mice with single infections, the recovery observed were equivalent to 5.75 log for groups infected with *C*. *albicans*, 6.18 log for groups infected with *C*. *krusei* and 4.33 log for groups infected with *C*. *glabrata*. These findings are consistent with Takakura et al. [24], where the same experimental models were used, recovering 5.70 log of *C*. *albicans* 3 days following inoculation. Costa et al. [39] also found 5.77 log of *C*. *albicans* 3 days following inoculation in immunosuppressed mice.

The present study is pioneering in documenting the development of oral candidiasis by *C*. *krusei* and *C*. *glabrata* in immunosuppressed mice models with single infections. *C*. *krusei* presented better ability to colonize the oral cavity of mice than the *C*. *albicans* species. This may be due to the fact that *C*. *krusei* also presents a better ability of agglutination in the presence of saliva when compared with C. *albicans*, thus accelerating the growth and development of biofilm [13, 40]. Even though all 3 species studied were able to colonize the oral cavity of mice, only *C*. *albicans* species was able to develop candidiasis, evidenced by macroscopic and microscopic lesions on the tongue dorsum. The ability of non-*albicans* species (*C*. *parapsilosis* and *C*. *tropicalis)* to colonize and cause diseases in a model host was studied by Mellado et al. [41], in which a murine model of gastrointestinal candidiasis was used. Although both species were successful in colonizing the gastrointestinal tract of the animals tested, *C*. *parapsilosis* and *C*. *tropicalis* displayed lower virulence when compared with *C*. *albicans* [41].

By examining the colonization profile of *C*. *albicans* in single and mixed infections, it became apparent that CFU/mL measurements, as well as the development of microscopic and macroscopic candidiasis lesions on the tongue dorsum of mice, were both considerably lower in multi-species infections compared to what was found in monospecies infections. However, extensive research is still required to ascertain the mechanisms by which *Candida* species compete amongst themselves during the onset of infection. Presumably, non-*albicans* species compete with *C*. *albicans* through areas of adherence to oral tissue, suppressing the formation of hyphae by *C*. *albicans*, or secreting substances that have antimicrobial action on other species. According to Thein et al. [13], if indeed non-*albicans Candida* species produces chemicals messengers that inhibit *C*. *albicans* filamentation, the recognition of such molecules should lead the way for the discovery of novel drugs for the treatment of candidiasis.

Further studies are also required to investigate the interactions among *Candida* species using a larger number of *Candida* strains. This study was performed only with laboratory reference strains and these findings need to be extended for clinical isolates. Previous studies demonstrated that the clinical isolates of *Candida* exhibit intra-species variability in relation to biofilm forming ability and to pathogenicity in animal models [36, 42–43].

From the evidence presented in this study, we concluded that *C*. *albicans* showed lower number of CFU/mL in mixed biofilms with non-*albicans* species when compared to single biofilm. In addition, single infections by C. *albicans* were more harmful for the animal models than mixed infections in association with *C*. *krusei* or *C*. *glabrata*. Thus indicating that *C*. *albicans* are able to establish competitive interactions with non-*albicans* species during biofilm formation and development of infection. Furthermore, the findings derived from studies on G. *mellonella* were consistent with the results found in the studies on mice, demonstrating the potential this invertebrate model has as an alternative to *in vivo* studies of microbial interactions.
